# Early Salience Signals Predict Interindividual Asymmetry in Decision Accuracy Across Rewarding and Punishing Contexts

**DOI:** 10.1002/hbm.70072

**Published:** 2024-11-25

**Authors:** Sean Westwood, Marios G. Philiastides

**Affiliations:** ^1^ University of Glasgow Glasgow Scotland

**Keywords:** decision, EEG, learning, punishment, pupillometry, reward, salience

## Abstract

Asymmetry in choice patterns across rewarding and punishing contexts has long been observed in behavioural economics. Within existing theories of reinforcement learning, the mechanistic account of these behavioural differences is still debated. We propose that motivational salience—the degree of bottom‐up attention attracted by a stimulus with relation to motivational goals—offers a potential mechanism to modulate stimulus value updating and decision policy. In a probabilistic reversal learning task, we identified post‐feedback signals from EEG and pupillometry that captured differential activity with respect to rewarding and punishing contexts. We show that the degree of between‐context distinction in these signals predicts interindividual asymmetries in decision accuracy. Finally, we contextualise these effects in relation to the neural pathways that are currently centred in theories of reward and punishment learning, demonstrating how the motivational salience network could plausibly fit into a range of existing frameworks.

## Graphical abstract

Post‐feedback salience signals in EEG and pupil data show clear differences in rewarding versus punishing contexts. The magnitude of these differences tracks the degree of cross‐context asymmetry in decision accuracy at the individual level.

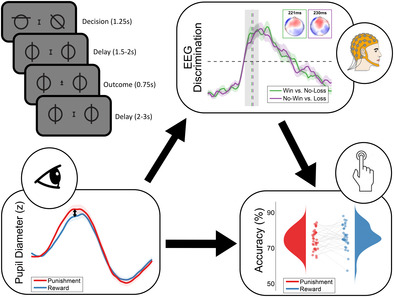

**Summary**

- Post‐feedback salience signals in EEG and pupil data show clear differences in rewarding versus punishing contexts.
- The magnitude of these differences tracks the degree of cross‐context asymmetry in accuracy at the individual level.
- The proposed salience signal is mechanistically compatible with a range of current theories of punishment learning.

## 1 Introduction

The classical account of instrumental learning dictates that actions leading to favourable outcomes will be reinforced, while actions leading to unfavourable outcomes will be diminished (Skinner 1938; Thorndike 1911). This basic principle of reinforcing behaviour has typically been understood through the reward prediction error (RPE) hypothesis, whereby the difference between expected and received outcomes is computed by phasic firing of midbrain dopamine (DA) neurons (Bayer and Glimcher 2005; Glimcher 2011; Schultz, Dayan, and Montague 1997). The dopaminergic RPE in this framework acts as a ‘teaching signal’ that updates an internal value representation for a given stimulus following an associated outcome (Hollerman and Schultz 1998), enabling the actor to better select for rewarding behaviours.

More recently, it has been proposed that an early unselective salience signal precedes the later RPE and value updating response independent of feedback valence or value (Schultz 2016). The concept of salience is broadly defined as the degree of bottom‐up attention attracted by a stimulus (Bordalo, Gennaioli, and Shleifer 2012, 2022), which can incorporate a variety of factors such as sensory intensity, novelty, surprise and relevance to motivational goals. With respect to the temporal dynamics of the dopaminergic RPE signal, there is evidence that adjusting different aspects of salience causes changes in the early response to stimulus presentation regardless of reward contingencies. For instance, the early activation of dopaminergic neurons has been shown to be diminished by reduced visual intensity (Tobler, Dickinson, and Schultz 2003) and reduced novelty through repeated exposure (Schultz 1998). Similarly, dopaminergic neurons show substantial activation to non‐rewarding stimuli only in the context of a reward‐rich environment (Kobayashi and Schultz 2014), implying that the degree of potential goal‐relevance (motivational salience) also contributes to the salience response.

In human neuroimaging, a similar two‐component (i.e., early/late) response has been observed with electroencephalography (EEG) during reinforcement learning (Philiastides et al. 2010). Subsequent EEG work with simultaneous functional magnetic resonance imagining (fMRI) supported this finding (Carvalheiro and Philiastides 2023; Fouragnan et al. 2015, 2017; Fouragnan, Retzler, and Philiastides 2018) and showed that the early component of feedback processing was related to regions including the anterior insula (aINS) and anterior cingulate cortex (ACC; Fouragnan et al. 2015), which are key areas within the so‐called salience network (Seeley 2019). The late component, on the other hand, involved areas traditionally implicated in reward and value processing, such as the ventral striatum (vSTR; Bartra, McGuire, and Kable 2013; Clithero and Rangel 2014; O'Doherty et al. 2004; Pagnoni et al. 2002) and ventromedial prefrontal cortex (vmPFC; Bartra, McGuire, and Kable 2013; Clithero and Rangel 2014; Gläscher, Hampton, and O'Doherty 2009). Furthermore, it was found that this later value signal was downregulated by the early salience signal (Fouragnan et al. 2015), indicating a modulatory effect of outcome salience on value processing and raising clear parallels to the midbrain dynamics outlined by Schultz (2016).

A key aspect of learning that the two‐component hypothesis may help to illuminate is the nature of learning in rewarding versus punishing contexts. This is due to the idea that individuals can have differing responses to these environmental conditions depending on their sensitivity to the goal of gaining reward versus the goal of avoiding punishment (McNaughton and Corr 2008), which would alter the motivational salience of feedback in each of these contexts and perhaps explain individual asymmetries in learning. Evidence from human neuroimaging has shown that certain regions such as the locus coeruleus (LC), aINS and vSTR show particularly distinct activation patterns in rewarding versus punishing contexts (Carvalheiro and Philiastides 2023; Palminteri et al. 2015), indicating the potential for highly variable individual dynamics in response to different types of reinforcer.

A prominent mechanistic account of punishment learning is that the RPE mechanism incorporates aversive feedback as a negative signal via the suppression of dopaminergic firing, similar to the unexpected omission of reward (Mirenowicz and Schultz 1996; Ungless, Magill, and Bolam 2004). If this account is accurate, a modulatory salience component could plausibly act via the habenula, which has been directly implicated in the processing of motivational salience (Bromberg‐Martin, Matsumoto, and Hikosaka 2010a, 2010b; Danna, Shepard, and Elmer 2013; Fakhoury and Domínguez López 2014; Hikosaka 2010), and seems influential for encoding aversive events and driving avoidance behaviour (Hennigan, D'Ardenne, and McClure 2015; Lawson et al. 2014; Lecca et al. 2017; Mondoloni, Mameli, and Congiu 2022). Importantly, the habenula has an inhibitory projection to dopaminergic activity in the ventral tegmental area (VTA) and substantia nigra (Christoph, Leonzio, and Wilcox 1986; Hikosaka 2010; Matsumoto and Hikosaka 2007), suggesting compatibility between the early salience hypothesis and this shared‐mechanism account of punishment learning.

However, some prominent findings have shown that distinct subpopulations of DA neurons in the midbrain show phasic excitation to aversive stimuli rather than inhibition (Brischoux et al. 2009; Cohen et al. 2012; Matsumoto and Hikosaka 2009). Additionally, certain studies have found no effects of pharmacological DA agents on punishment learning, despite significant concurrent effects on reward learning (Eisenegger et al. 2014; Jocham, Klein, and Ullsperger 2011; Pessiglione et al. 2006; Rutledge et al. 2009). This could suggest that punishment learning depends on a specific punishment prediction error (PPE) signal rather than a common RPE signal (Palminteri and Pessiglione 2017). If this is the case, an early salience signal as presented by Fouragnan et al. (2015) is also compatible with many of the regions shown to exhibit distinct activation to aversive feedback during learning, including aINS (Combrisson et al. 2023; Gueguen et al. 2021; Klavir, Genud‐Gabai, and Paz 2013; Palminteri et al. 2012, 2015) and ACC (Fujiwara et al. 2009; Klavir, Genud‐Gabai, and Paz 2013; Monosov 2017). Crucially, though it is not yet clear exactly how the reward–punishment dichotomy is processed in the brain, the most prominent accounts that have been proposed thus far seem to be mechanistically compatible with an early salience signal that modulates subsequent value processing, making this a plausible avenue for investigation. As such, possible salience effects can be examined from an agnostic position as to the core mechanism of reward and punishment encoding.

In this work, we aimed to investigate the extent to which we can differentiate interindividual learning propensities across the two contexts from neural and physiological measures. Specifically, we exploited an early salience electrophysiological (EEG) component, appearing at around 220 ms post‐feedback (Philiastides et al. 2010), that has previously been shown to emerge following reward omissions, with a subsequent downstream influence on a separate value processing stage (Fouragnan et al. 2015, 2017; Fouragnan, Retzler, and Philiastides 2018). This relationship is consistent with dual‐component dynamics observed in midbrain DA neurons, where an early salience response to feedback modulates a later value‐related signal (Schultz 2016). This could point to a general salience mechanism, compatible with any of the main theories of reward and punishment encoding, that forms a crucial initial stage of reinforcement learning in the brain and explains a degree of individual variability in behavioural responses.

Adapting the paradigm of Fouragnan et al. (2015) to include distinct rewarding and punishing contexts in a reversal learning task, we first aimed to identify EEG post‐feedback responses that are linearly separable across the two contexts independently for both positive and negative outcomes, leveraging the high temporal resolution to isolate the early salience‐related component. This approach allows us to have a direct valence comparison without any confounding effects of outcome sign, such that the unique distinguishing factor in each comparison is whether the outcomes are relevant to a reward‐ or punishment‐related outcome. Subsequently, we investigated whether these representations are consistent with the early salience signals reported in previous studies and test the extent to which they explain interindividual asymmetries in behaviour across contexts. Since there is evidence that the LC has both distinct contextual dynamics across reward and punishment as well as functional connectivity to key salience areas (Carvalheiro and Philiastides 2023), and this nucleus is known to drive phasic pupil dilation (Larsen and Waters 2018; Mathôt 2018), we also used phasic pupil dilation as an indirect proxy measure to test how differences in LC‐driven noradrenergic activations relate to EEG‐derived salience representations and whether they further explain subject‐specific behavioural changes across contexts.

## 2 Materials and Methods

### 2.1 Participants

**FIGURE 1:**
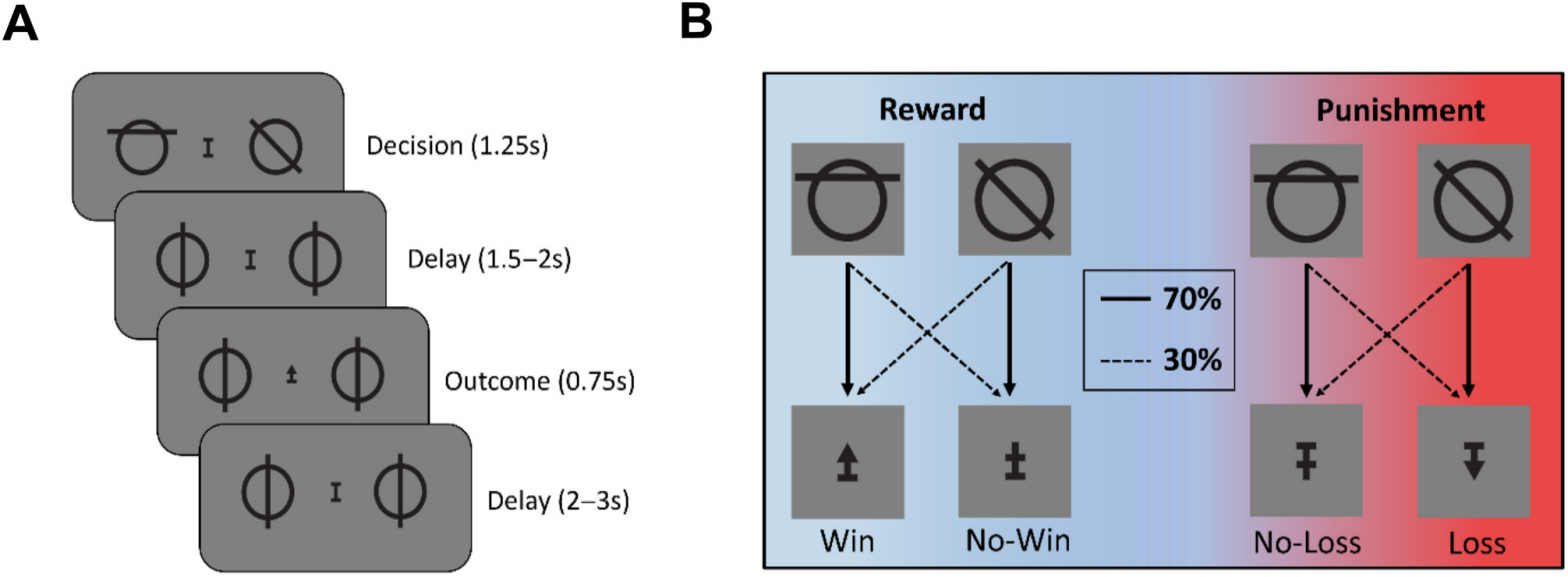
Depiction of probabilistic reversal learning task. (A) Stages of a single trial. Participants choose one of two symbols with a button press for a maximum of 1.25 s. If no choice was provided in this time, the message ‘Please respond faster’ was displayed. After a short delay, the outcome is presented in the centre of the screen. (B) Outcome symbols and contingencies. Participants always choose between the same two symbols throughout the entire task. For a given trial, one of these symbols has a 70% chance of a positive outcome, while the other has a 30% chance. In the appetitive condition, a positive outcome is the ‘win’ symbol and a negative outcome is the ‘no‐win’ symbol; in the aversive condition, a positive outcome is the ‘no‐loss’ symbol and a negative outcome is the ‘loss‘ symbol. These contingencies switch approximately every 20 trials during an 80‐trial block.

**FIGURE 2:**
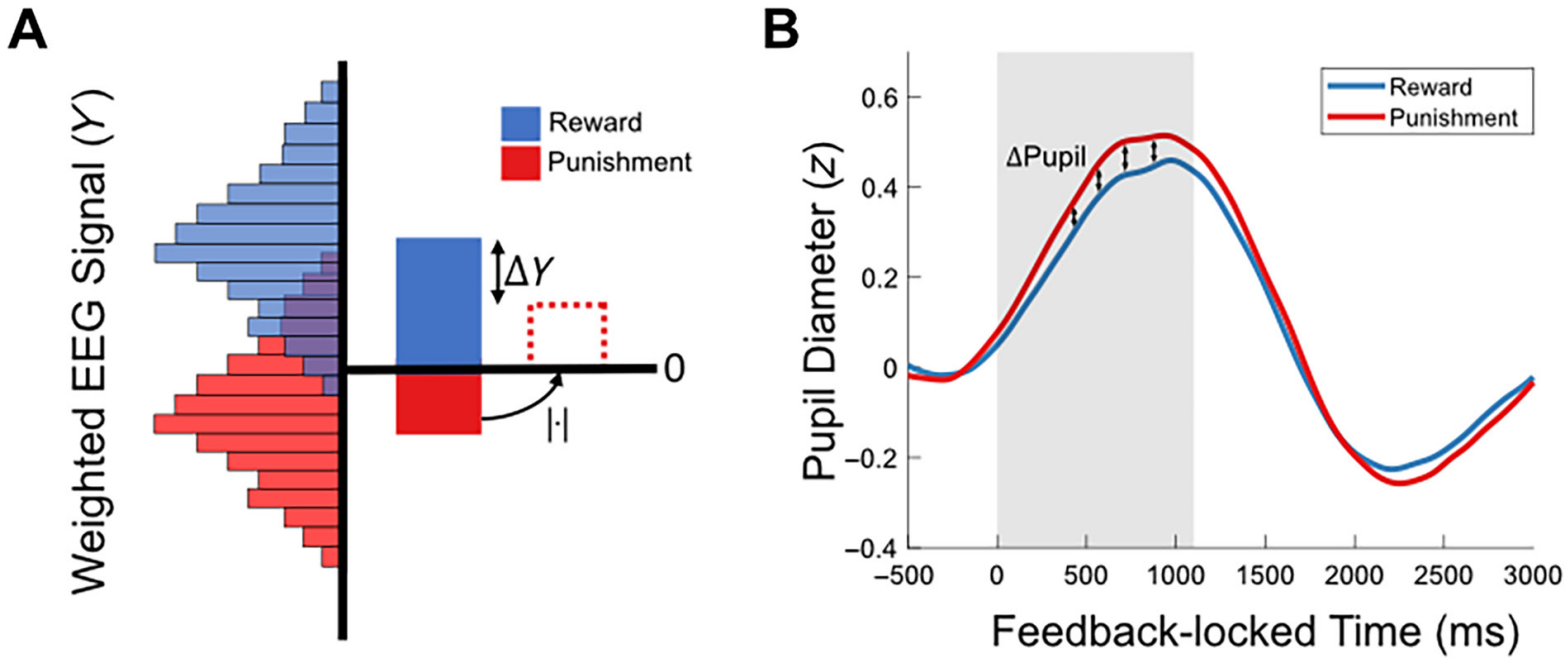
(A) Depiction of Δ*Y* measure for a hypothetical participant. Histograms show trial‐by‐trial distribution of weighted EEG (*Y*) values from the multivariate discrimination (as defined in Equation 1) across reward (blue, upper) and punishment (red, lower) trials. For the positive‐outcome model, reward trials reflect wins and punishment trials reflect non‐losses. For the negative‐outcome model, reward trials reflect no‐wins and punishment trials reflect losses. Solid bars show mean *Y* value averaged across trials. Dotted red bar shows conversion of mean punishment *Y* to absolute value for subtraction from the mean reward *Y*. (B) Depiction of Δpupil measure for an example participant. Blue and red lines show mean *z*‐scored pupil response post‐feedback for reward and punishment trials respectively. Single Δpupil measure is computed by averaging over the shaded area—which highlights the window of significance from the non‐parametric clustering test—for each condition and subtracting the punishment value from the reward value. This procedure is carried out separately for positive‐ and negative‐outcome trials.

**FIGURE 3:**
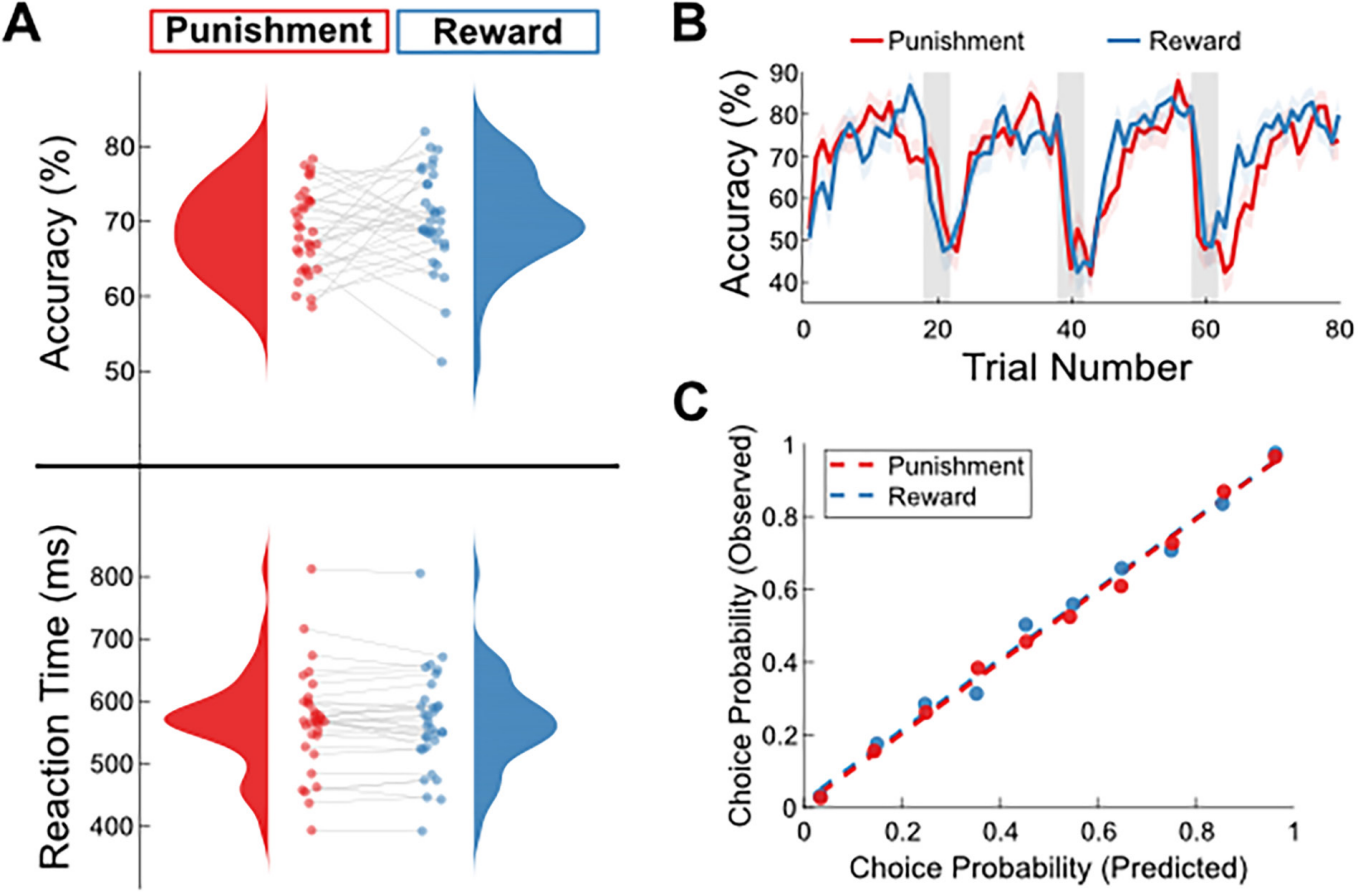
(A) Comparison of choice accuracy (upper panel—percentage chosen for high‐value symbol) and reaction time (lower panel—time from symbol presentation to choice in milliseconds) across reward and punishment conditions. Blue (right side of each plot) scatters show individual data points for reward context, while red (left side of each plot) show equivalent data for punishment context. (B) Percentage of high‐probability symbol chosen for each trial across a block, averaged across blocks and participants separately for reward (blue) and punishment (red) contexts. Shaded areas indicate trials where a reversal can occur and pupil data from positive outcomes, and right depicts the same for negative outcomes. (C) Reinforcement learning model performance for reward (blue) and punishment (red) trials. *X*‐axis represents model‐derived choice probabilities for a given symbol binned into deciles for each subject and averaged across subjects. *Y*‐axis represents proportion of corresponding trials in each bin where that symbol was chosen, averaged across subjects.

Data were collected from 33 participants, (18 female, 15 male) with ages ranging from 18 to 41 (mean = 23.30 years, SD = 5.29). We excluded six participants from pupil analyses (Figure 1A,B) due to excessive missing data in the pupil recording, defined as > 47% of samples missing for reward blocks and > 51% for punishment blocks (based on one standard deviation from the mean). We also excluded one participant from EEG analyses (Figure 1C) due to excessive movement artefacts in the EEG signal. For the combined EEG and pupil analyses (all linear regressions and subsequent mediation analysis, Figures 2 and 3) this left 26 remaining participants with usable data for both EEG and pupillometry (14 female, 12 male, mean age = 23.15 years, SD age = 5.87). All participants were recruited through the University of Glasgow Subject Pool, were right‐handed and had uncorrected vision. The study was approved by the College of Science and Engineering Ethics Committee at the University of Glasgow and informed consent was obtained from all participants.

### 2.2 Task and Procedure

**FIGURE 4:**
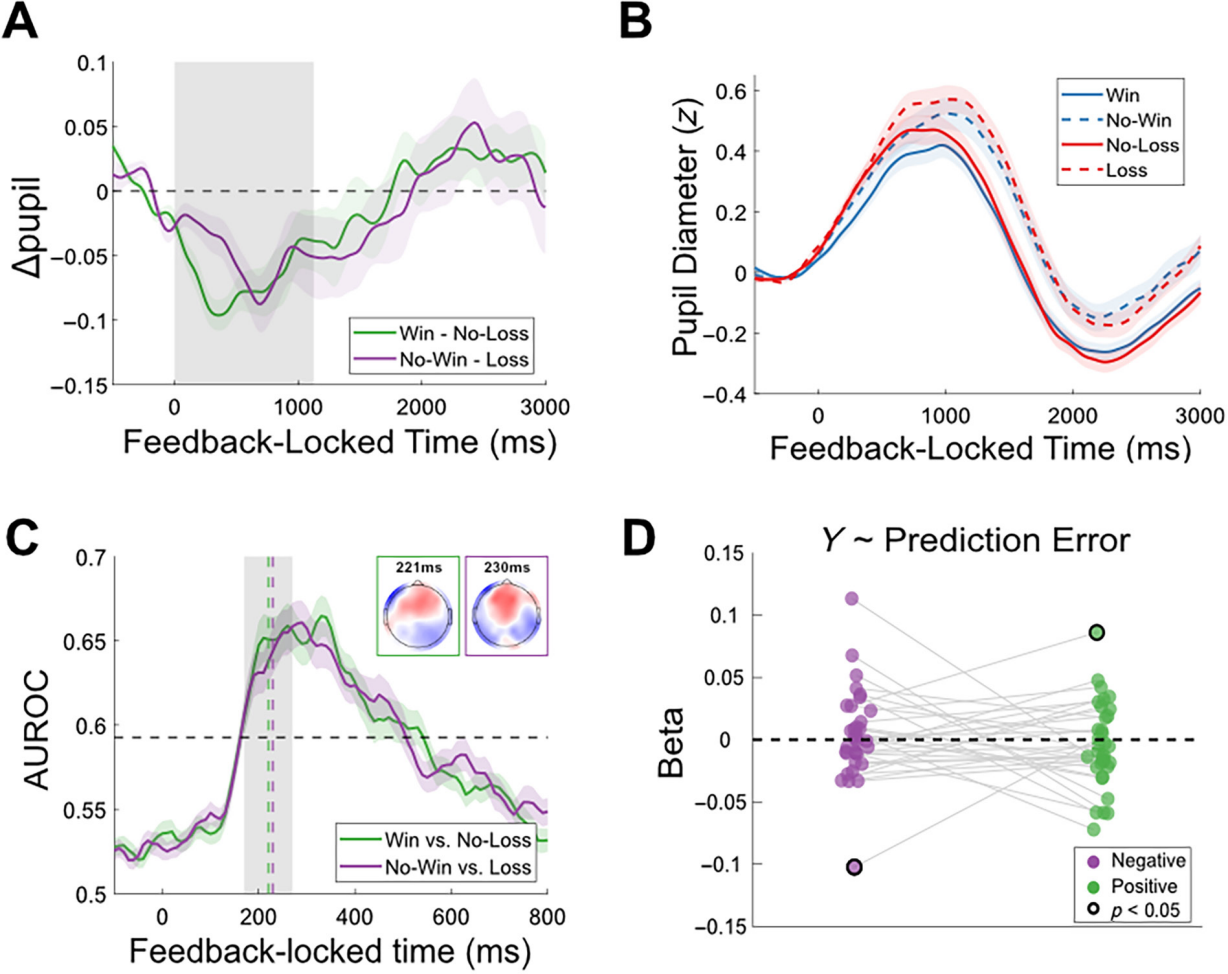
(A) Difference score (reward–punishment) of the post‐feedback pupil signal averaged across participants separately for positive outcomes (win − no‐loss, green) and negative outcomes (no‐win − loss, purple). Shaded area indicates window of significant difference between pupil response in reward versus punishment conditions averaged across all trials, obtained from non‐parametric cluster test. (B) Post‐feedback pupil response averaged across trials and participants, separated by positive (solid line) and negative (dotted line) outcomes. Red indicates punishment condition and blue indicates reward condition. *X*‐axis represents time from feedback onset in milliseconds and *y*‐axis represents *z*‐scored pupil diameter. (C) AUROC (area under receiver operating characteristic curve) values and scalp topographies for two separate classification models. *Y*‐axis depicts mean feedback‐locked area under AUROC for logistic regression averaged across subjects. *X*‐axis depicts time from feedback onset in milliseconds. Shaded error bar represents standard error of the mean across subjects. Grey shaded area reflects window for peak selection, and dotted vertical lines depict average peak onset for positive (win vs. no‐loss, green) and negative (no‐win vs. loss, purple) outcomes. Scalp topographies show average forward model from subject‐specific peaks—conditioned were arbitrarily mapped as negative (red) for punishment and positive (blue) for reward. Horizontal dashed line depicts *p* = 0.01 permuted significance threshold averaged across subjects and across the two classification models. (D) Beta coefficients for individual participants from a linear model predicting trial‐by‐trial *Y* amplitudes from unsigned prediction error from the reinforcement learning model. Purple dots (left) show coefficients from negative‐outcome trials only, and green dots (right) show coefficients from positive‐outcome trials only. Black outline indicates the beta coefficient value for that subject was significant.

The study used a simple probabilistic reversal learning paradigm, based largely on the design used in Fouragnan et al. (2015) with the addition of a reward–punishment manipulation (Figure 4). The main task consisted of six blocks of 80 trials, alternating between rewarding and punishing contexts. We decided to always start with a rewarding block rather than counterbalancing the order, as we wanted to maximise the feeling of earning money and then subsequently losing it to increase the subjective difference between the contexts. Each trial began with a jittered 2–3 s fixation period before the decision phase, after which participants had to choose between two symbols with mirrored probabilities (70% and 30%) of a positive or negative outcome, and the same pair of symbols was used in every trial throughout the whole task. Outcomes for the symbols on a given trial were independent of each other, meaning that on a given trial it is possible for both symbols to yield the same outcome. If the participant did not respond within 1.25 s of the decision phase, they were informed that they would lose £0.50 and the message ‘Please Respond Faster’ was displayed. The decision phase was followed by a jittered delay period lasting between 1.5 and 2 s, before a 0.75 display of the outcome symbol. Participants indicated their choice via left or right button press on a specialised response box (Cedrus RB‐740 Response Pad, Cedrus, USA). We provided positive and negative outcomes by displaying different arrows in the centre of the screen. Specifically, in reward blocks, we used upward and neutral arrows to respectively provide positive and negative feedback, and in punishment blocks we used neutral and downwards arrows to respectively provide positive and negative feedback. To minimise pupil fluctuations due to visual properties, all arrows and fixation symbols were normalised for perceptual load and luminance using consistent pixel count and geometric structures, and transitions between fixation, decision and outcome screens were kept as subtle as possible. If a participant did not respond in time, a brief ‘too slow’ message appeared on the screen before the next trial, which participants were informed would carry a penalty of −£0.50 to disincentivise missed trials.

Participants aimed to ascertain once the symbol with the 70% probability of success, participants could select it repeatedly to maximise their monetary payout. However, the outcome contingencies of the symbols would switch approximately every 20 trials (±2), such that the ‘good’ option would become the ‘bad’ option and vice versa. The participants were told these switches would occur ‘every so often’ throughout each block, but both the outcome contingencies and reversal frequencies were not known to the participant. Therefore, following unexpected outcomes, participants had to infer whether this was due to inherent stochasticity in the design or a change in the underlying contingencies. This task design was chosen to provide a simple reinforcement learning paradigm with clear truth labels for correct choice and a steady degree of volatility to allow for a variety of decision‐making strategies, as well as to provide consistency for comparison with similar studies such as Fouragnan et al. (2015).

Participants were paid a baseline of £10 for participation and could additionally earn between £5 and £20 based on task performance. This was implemented by adding £0.25 to the total reward for each ‘win’ outcome and subtracting £0.25 for each ‘lose’ outcome, while no‐win and no‐loss outcomes yielded £0. The total amount won or lost was displayed after each block to keep the participant engaged with the consequences of the outcome symbols, and the experimenter reminded participants whether the upcoming block was rewarding or punishing. The average total reward was approximately £20 for a 2.5‐h session (including baseline).

Before attending, all participants completed a shorter online practice version of the task, which was implemented using Pavlovia, an online version of PsychoPy (Peirce et al. 2019). A minimum of 60% accuracy over 96 trials was required for participation.

### 2.3 EEG Data Collection and Analysis

We sampled data at 1000 Hz from a 64‐channel EEG cap (BrainCap, BrainProducts, Germany) and accompanying amplifiers (BrainAmp, BrainProducts, Germany), using the Brain Vision Recorder software (BVR, Version 1.2.1 BrainProducts, Germany). The Ag/AgCl electrodes were positioned according to the international 10–20 system and all electrodes referenced to the left mastoid, with a ground electrode positioned on the left mandible. All electrode impedances were kept below 20 kΩ using conductive gel. The amplifiers had a built‐in hardware band‐pass filter of 0.0016–1000 Hz. We applied a band‐pass filtered to the data using a 0.5 Hz Butterworth high‐pass filter to remove slow direct current drifts and a 40 Hz Butterworth low‐pass filter to remove higher frequencies of no interest. To remove eye‐blink and ‐movement artefacts, participants performed an eye calibration task before the main experiment during which they were instructed to blink continuously for several seconds, and then track a cross moving horizontally and vertically while keeping their head still. We recorded the timing of these events and used principal component analysis (Parra et al. 2005) to identify linear components associated with eye‐blinks and ‐movements, which we subsequently projected out of the broadband EEG data collected during the main task.

For each participant individually, we employed a multivariate discrimination analysis on the EEG signal, whereby an optimal set of electrode weights was estimated using a logistic regression model to maximally discriminate between trials from the reward condition and trials from the punishment condition separately for positive outcomes and negative outcomes. This analysis was designed to address our first hypothesis, that there would be observable differences in early salience signals between the reward and punishment contexts. The outcomes were analysed separately to isolate context effects and avoid interactions from the outcome valence signals that have been observed in previous studies (Fouragnan et al. 2015, 2017). In one analysis ‘win’ trials were discriminated against ‘no‐loss’ trials, and in the other analysis ‘no‐win’ trials were discriminated against ‘loss’ trials, employing a method based on Parra et al. (2005) and Sajda et al. (2007). Though positive outcomes had higher trial counts, the numerical discrepancy for positive versus negative trials was < 15% for all participants and < 10% for the vast majority, with the largest difference being 258 positive outcomes versus 222 negative outcomes. We applied a sliding 60 ms window in 10 ms increments from 100 ms pre‐feedback to 800 ms post‐feedback, and within each window data were used to train a logistic regression model, where outcomes in the rewarding context (i.e., wins and no‐wins) were arbitrarily mapped to positive values and punishments (i.e., losses and no‐losses) to negative values relative to the discriminating hyperplane. Each electrode represented one predictor variable in the model, resulting in 64 weightings *w* that optimally predicted context depending on the analysis. When applied to the EEG signal X, the resulting weighted amplitudes could be summed across electrodes to produce a single scalar component amplitude Y, representing linear distance from the discriminating hyperplane:

(1) $$Y\left(t\right)=w^{T}\cdot X\left(t\right)$$

To visualise the spatial representation of the resulting discriminating components, we calculated a forward model which captures the relative contribution of each sensor to the discrimination (note all topographies shown in the paper depict this forward model):

(2) $$a=\frac{X\cdot Y}{Y^{T}\cdot Y}$$

Discriminator performance was quantified using the area under a receiver operating characteristic curve (AUROC) using a leave‐one‐out cross‐validation approach. To assess the significance of these AUROC values across time, we used a permutation approach whereby a null AUROC distribution was derived from 1000 permutations of the same classifier with randomly shuffled labels for reward and punishment, and a significance threshold was set at the 99th percentile (*p* < 0.01). We identified AUROC peaks for each participant separately, representing the point of individual maximum AUROC value between 170 and 270 ms, which corresponds to the early salience‐related signal outlined in the dual‐component theory of feedback processing, encompassing ±50 ms from previous findings (Fouragnan et al. 2015; Philiastides et al. 2010). To avoid our early salience peaks being selected on the upward slope of a subsequent value‐related peak (as found in (Fouragnan et al. 2015)), we only considered for peak selection time‐points where the AUROC value was greater than that of the two preceding and two following time‐points—in other words, a local maximum. These subject‐specific peaks were then used to extract the corresponding *Y* value for use in subsequent analyses.

### 2.4 Pupillometry Data Collection and Analysis

Pupil diameter and gaze *x*/*y* coordinates were recorded at 40 Hz using a screen‐based eye‐tracker (Tobii Pro X3‐120, Tobii, Sweden). All stimuli were made with equivalent pixel counts to ensure equiluminance and were designed to minimise shape change between screens to minimise light‐related pupil fluctuations.

Missing pupil data due to blinks was addressed by linearly interpolating samples within ±100 ms of blink events. We then applied a band‐pass filter of 0.01–4 Hz, *z*‐scored the resulting data, and epoched each trial to −500/+2000 ms around feedback, baseline corrected to 500 ms pre‐feedback. Outlier trials for each subject were identified as > 3 standard deviations from the mean (averaged across trials and samples over the epoched window), or < 1.5% of mean variance (variance calculated across time and averaged across trials). The latter was specifically to deal with occasional flat lines in pupil response due to errors at data collection. All outlier trials were then removed before any further analysis, averaging at 9.58 trials removed per participant.

To determine a difference in pupil response between contexts as per our first hypothesis, we used a non‐parametric approach based on the single‐sensor time‐series analysis outline by Maris and Oostenveld (2007). An independent *t*‐test between reward and punishment contexts was conducted for each time‐point across subjects, and with the non‐parametric test statistic being the sum of *t*‐values for the largest cluster of consecutive significant results, falling between 0 and 1100 ms post‐feedback. We then compared the resulting test statistic (df = 26, ∑*t* = 180.01) to the 99th percentile of 10,000 permutations of test statistics from randomly allocated groups (df = 26, ∑*t* = 6.30) to determine statistical significance (Figure 1A).

### 2.5 Computational Modelling

We trained a model‐free reinforcement learning algorithm on trial‐by‐trial choices for each subject. This functions by estimating for trial t an RPE $\delta_{t}$ from the difference in expected value $V_{t}$ and received reward $r_{t}$ of choice i:

(3) $$\delta_{t}=r_{t}-V_{t}\left(i\right)$$

This principle is then used to update expected value by weighting this RPE with a learning rate parameter α. This parameter lies between 0 and 1, with a greater learning rate implying a faster updating of value expectations based on recent evidence:

(4) $$V_{t+1}\left(i\right)=V_{t}\left(i\right)+\alpha \cdot \delta_{t}$$

To account for fluctuations in perceived environmental volatility, the learning rate parameter was also dynamically updated via the slope of the smoothed RPE m as outlined in (Krugel et al. 2009):
(5)

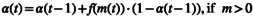



(6)

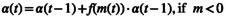




Here, $f\left(m\left(t\right)\right)$ is a double sigmoid function that transforms m such that 0<m<1, which then scales the trial‐wise dynamic learning rate. This function recruits an additional free parameter, which reduces the degree to which alpha is modulated as it increases.

Finally, choice probability for a given choice i was derived according to a softmax decision rule, which adds an additional parameter for inverse temperature γ (temperature being the degree of stochasticity in decisions, represented by the slope of the sigmoid):

(7) $$p_{i}\left(t\right)=\frac{e^{\gamma \cdot V_{i}\left(t\right)}}{\sum_{j=1}^{n}e^{\gamma \cdot V_{j}\left(t\right)}}$$

### 2.6 Subject‐Specific Context Sensitivity

Our main aim was to test whether differences in neural or pupil signals between contexts can predict corresponding behavioural asymmetries across participants. Going forward, these comparisons will be referred to with the Δ prefix, which in all cases indicates the punishment condition subtracted from the reward condition for a given measure. The primary behavioural measure of context sensitivity is Δaccuracy, which is simply the proportion of correct choices attained in the punishment context subtracted from the proportion of correct choices attained in the reward context. As such, as positive value for Δaccuracy indicates greater average accuracy in the reward context. A correct choice refers to trials where the symbol with higher probability of reward or punishment omission was chosen.

**FIGURE 5:**
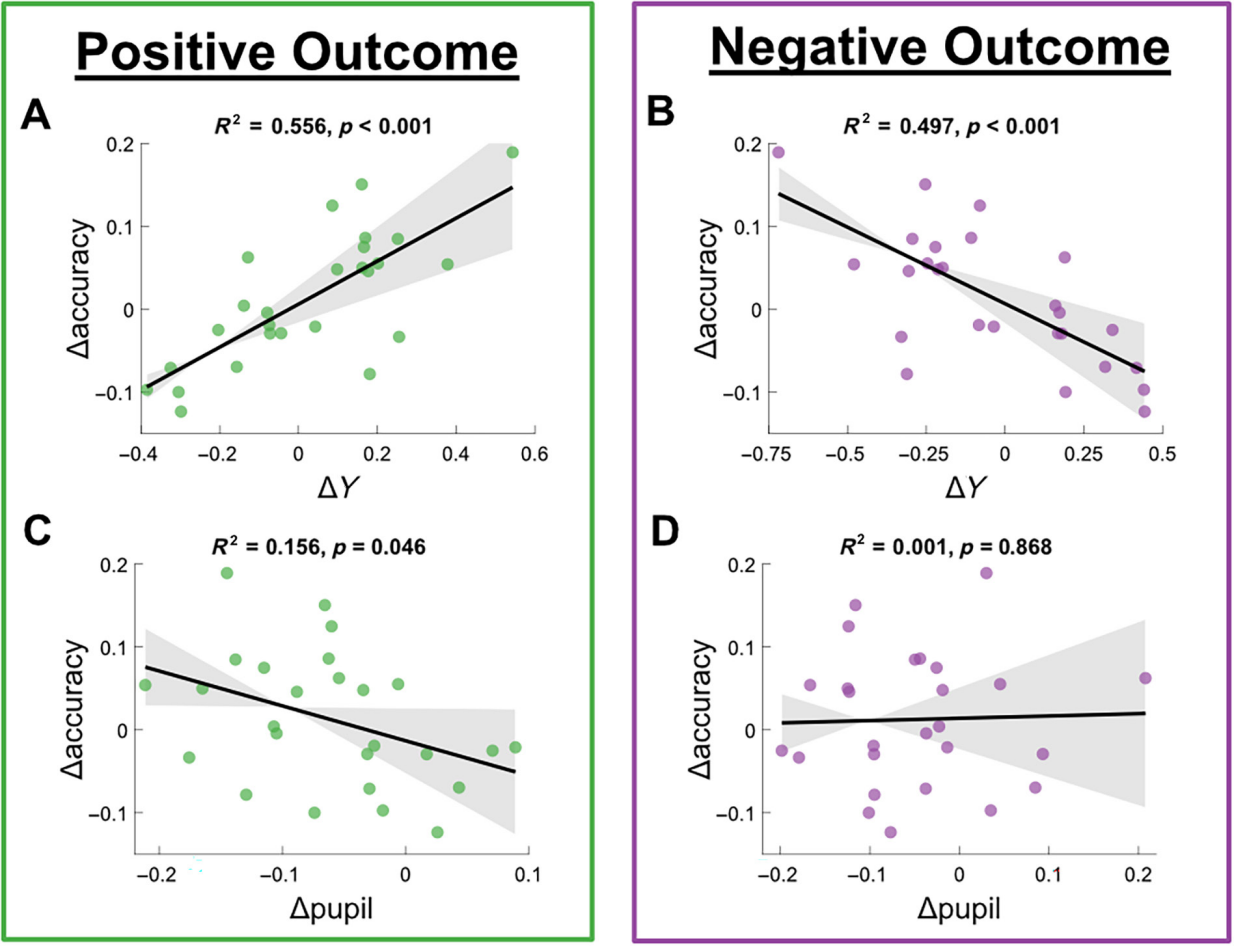
(A and B) Δaccuracy linearly predicted by Δ*Y* across subjects. Shaded error bars indicate 95% confidence intervals for the estimate. Δ‐accuracy is the same measure calculated across all trials for all plots, whereas Δ*Y* is separated by classification model trained on positive‐outcome (left, green) and negative‐outcome (right, purple) trials. Positive value on *x*‐axis indicates that EEG data for reward condition is on average further from the discriminating hyperplane than EEG data for punishment condition in a given participant, and vice versa. Positive value on the *y*‐axis indicates higher proportion of correct choices in reward condition versus punishment condition for a given participant. (C and D) Equivalent plots with Δpupil (reward–punishment) depicted on the *x*‐axis rather than EEG components. Again, Δaccuracy is identical across both plots, whereas Δ‐pupil is separated by outcome type.

Given that the EEG‐derived *Y* measurement reflects the distance from the discriminating hyperplane towards either the rewarding or punishing context, Δ*Y* is designed to show the average asymmetry in neural signals across contexts. For positive and negative outcomes separately, Δ*Y* for an individual participant is calculated by subtracting the absolute mean *Y* magnitude for punishment condition trials from the absolute mean *Y* magnitude for reward condition trials (Figure 5A). For example, a Δ*Y* value greater than 0 for positive outcomes would indicate that on average, for an individual participant, the neural signal induced by reward more pronounced and distinct than the neural signal induced by punishment omission.

We leveraged the non‐parametric window of significance (0‐1100 ms as outlined in the previous section) to calculate a Δpupil score, where mean pupil amplitude across the window in the punishment context was subtracted from the reward context for each participant. We chose to average across the window rather than select a single value at the peak as our non‐parametric analysis demonstrated that many of the between‐context differences are not accounted for by differences at the peak alone. As with the Δ*Y* above, Δpupil was computed separately for positive‐ and negative‐outcome trials to avoid possible confounding effects of outcome (e.g., signals associated with error detection) on the pupil diameter. A positive value for Δpupil would indicate that a participant exhibited greater phasic dilation in response to outcomes in the reward context compared to the punishment context. Taking the difference score here isolates context‐driven dilation effects by subtracting out common outcome‐related arousal responses.

Together, these Δ scores allow us to quantify the extent to which context‐dependent differences in EEG and pupil signals track context‐dependent asymmetries in task performance. We therefore leveraged these scores to test our second hypotheses using simple linear regression, specifically that across reward and punishment contexts, differences in LC‐driven pupil dilation and in discriminating EEG signals will predict subsequent asymmetries in behavioural accuracy.

### 2.7 Mediation Analysis

Our final hypothesis proposes that task performance is influenced by a salience signal visible in EEG data, which is in turn downstream of LC activation that drives pupil dilation. Because of the sequential nature of this hypothesis, a mediation analysis was used to determine whether the neural processes behind the Δ*Y* value facilitate a relationship between LC‐driven Δpupil and subsequent Δaccuracy. The goal of the mediation analysis is to identify whether the relationship between a predictor variable (Δpupil) and an outcome variable (Δaccuracy) can be explained by a mediator variable (Δ*Y*).

Typically for a mediation effect to be considered plausible here there are three preconditions: (1) the predictor variable (Δpupil) should significantly predict the outcome variable (Δaccuracy) in a simple linear regression; (2) the predictor variable (Δpupil) should significantly predict the mediator variable (Δ*Y*) in a simple linear regression; and (3) the mediator variable (Δ*Y*) should significantly predict the outcome variable in a simple linear regression (Δaccuracy) (Baron and Kenny 1986; Shrout and Bolger 2002). In some cases, condition (1) can be considered non‐essential, such as where the effects in (2) and (3) have opposite directions (MacKinnon, Krull, and Lockwood 2000). The mediation effect itself reflects the difference in predictive strength (the beta coefficient) of Δpupil on Δaccuracy in the simple regression model versus in the multiple regression model that includes Δ*Y* (VanderWeele 2016). For positive and negative outcomes separately, we used the M3 toolbox for Matlab (Wager et al. 2008; https://github.com/canlab/MediationToolbox) to establish the preconditions and significance of the mediation effect using a 10,000 sample bootstrap test on the resulting statistic (Wager et al. 2008).

## 3 Results

### 3.1 Behavioural Results and Model Fit are Similar across Reward and Punishment

Subjects displayed a high level of accuracy across both conditions of the task, choosing the high‐value symbol on average 70% of the time in the reward condition and 69% of the time in the punishment condition. At the group level, paired *t*‐tests revealed no clear behavioural differences in accuracy (df = 32, *t* = 1.20, *p* = 0.24) or reaction time (df = 32, *t* = −0.77, *p* = 0.45) between the rewarding and punishing contexts (Figure 3A). Participants on average displayed the typical learning patterns we expect in a reversal learning task, with choice accuracy drastically falling following a reversal before climbing back up as the new contingencies are realised (Figure 3B). Observed subject choices closely matched reinforcement learning model predictions for both reward and punishment trials (Figure 3C; *r* < 0.001).

**FIGURE 6:**
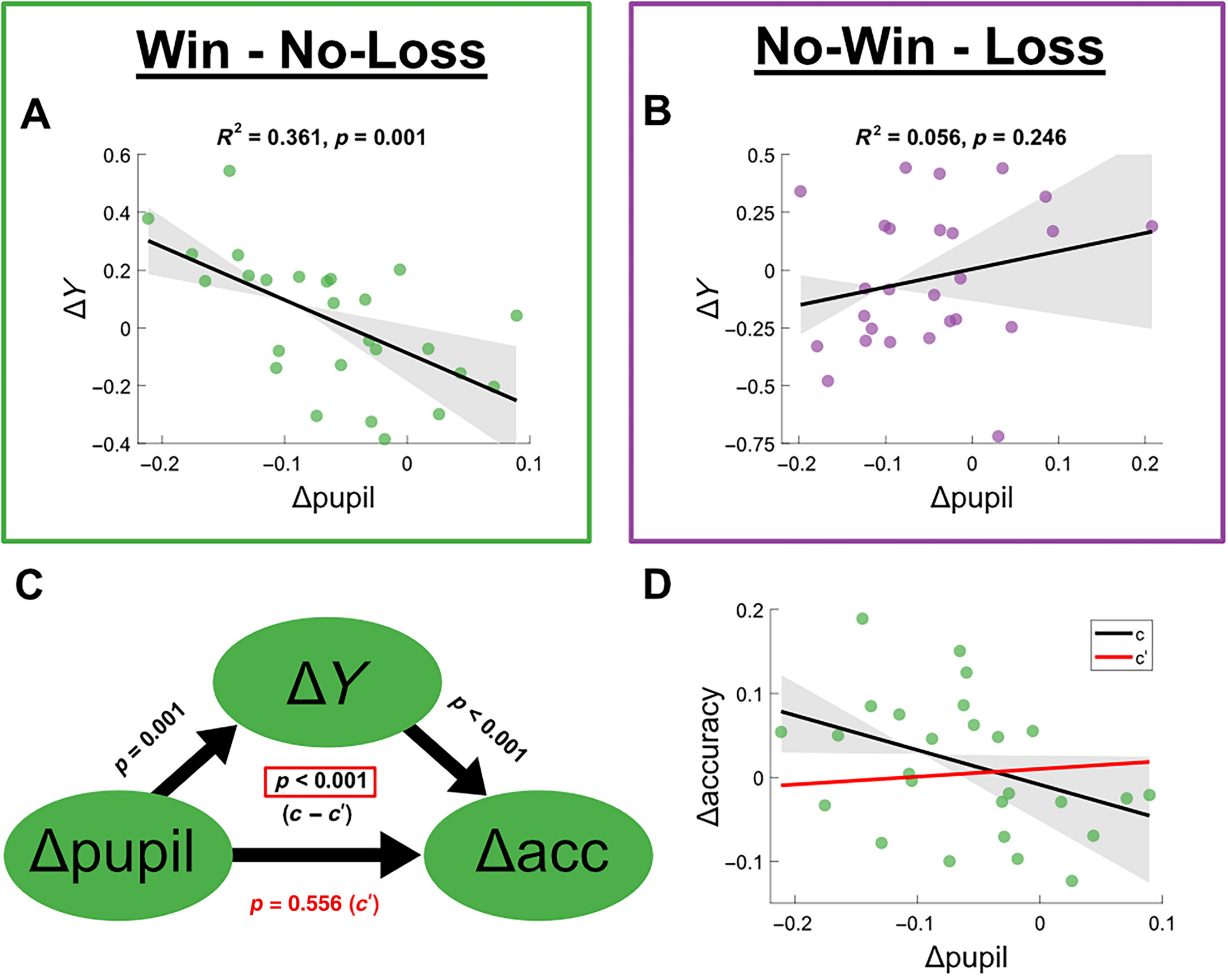
(A and B) Δ‐pupil correlated with ΔY across subjects. As in Figure 5, (A) shows a significant prediction of Δ*Y* from Δpupil for positive‐outcome trials, whereas (B) shows no significant relationship between the two for negative‐outcome trials. (C) Mediation analysis (for positive outcomes only) showing the effect of Δpupil on Δaccuracy with Δ*Y* as a mediating variable. *p* values indicate as follows: Left—linear prediction of Δ*Y* by Δpupil; Right—linear prediction of Δaccuracy by Δ*Y*; Bottom—direct effect of pupil change on accuracy change when ΔY is included as a predictor in a multivariate regression (c′; direct effect). Middle—permutation test of comparison of model coefficient for Δpupil predicting Δaccuracy when Δ*Y* is included as a predictor (c′; direct effect) versus not (c; total effect). (D) Depiction of the two coefficient lines c and c′ from the mediation analysis. The black line indicates the slope of the effect of Δpupil on Δaccuracy in a simple linear regression, as depicted fully in Figure 5C (*β* = −0.420, *p* = 0.046). The red line indicates the slope of the same effect in a model where Δ*Y* is included as an additional predictor (*β* = 0.089, *p* = 0.556).

### 3.2 Distinct EEG and Pupil Responses to Reward and Punishment Capture More Than Surprise

Pupil diameter post‐feedback followed a typical impulse response profile for all contexts and outcomes, however these factors parametrically affected deviation from pre‐feedback baseline. Negative outcomes elicited a greater dilation than positive outcomes, as did punishing contexts compared to rewarding contexts (Figure 1B). Our non‐parametric cluster test (Maris and Oostenveld 2007) revealed significant differences for each of these comparisons across the 0‐1100 ms window. This is depicted by the shaded area in Figure 1A, which contains the negative Δpupil signal (reward–punishment) separately for positive and negative outcomes. Taken alongside the EEG findings, this supports our first hypothesis that salience‐related signals will be significantly different across reward and punishment contexts.

To investigate whether any group differences emerged at the neural level, two single‐trial multivariate discriminant analyses were used on EEG data locked to the time of decision feedback to separate the reward and punishment contexts; one trained on trials where the outcome was positive, the other negative. Separability between reward and punishment context was significantly greater than 0.5 between 170 and 530 ms for positive‐outcome trials and 170 and 500 ms for negative‐outcome trials, determined by AUROC values that exceeded the significance threshold from a 1000‐sample permutation test (*p* < 0.05) (Figure 1C). A window of interest was set at 170–270 ms to isolate the early salience component from a later value updating component, based on timings from previous studies (Fouragnan et al. 2015; Philiastides et al. 2010). At the individual level, a subject‐specific discrimination peak was taken as the highest out of all AUROC values greater than the preceding and following two AUROC values within the specified window of interest. Averaged across participants, this yielded a component peak at 221 ms for positive outcomes, and 230 ms for negative outcomes (Figure 1C). The scalp topographies averaged across subjects at these moments reflected a similar fronto‐central cluster to that observed in previous early components (Fouragnan et al. 2015, 2017; Philiastides et al. 2010), and were highly comparable across the two discrimination analyses trained separately on positive and negative outcomes (Figure 1C; insets).

To test whether the EEG discrimination component was reflective of surprise, we used a linear regression to predict the trial‐wise discrimination component amplitudes (*Y*s) from unsigned prediction error derived from our computational reinforcement learning model (Figure 1D). A simple contrast analysis showed that for both positive (*t*(31) = −0.743, *p* = 0.462) and negative outcomes (*t*(31) = −0.603, *p* = 0.551), subject‐specific model coefficients were not statistically significant from zero, indicating that the EEG component amplitude contains information other than pure surprise at an outcome.

### 3.3 Accuracy Changes Across Contexts Are Tracked by EEG and Pupil Metrics

Despite similarities in behaviour across reward and punishment contexts at the group level (Figure 6A,B), there was significant interindividual variability in accuracy (Figure 6A), and clear differences in neural and physiological signals emerged. To address our second hypothesis and understand whether individual dynamics in accuracy were predicted by changes in EEG and pupil signals across contexts, we used simple linear regression to predict the individual Δaccuracy values across participants using the other Δ measures outlined in the methods section.

We found that Δaccuracy was strongly positively predicted by Δ*Y* for positive outcomes (Figure 2A; *R* ^2^ = 0.556, *F*(1,24) = 30.039, *p* < 0.001) and negatively predicted for negative outcomes (Figure 2B; *R* ^2^ = 0.497, *F*(1,24) = 23.671, *p* < 0.001). In each case, a discrimination component driven primarily by the polarised outcome (rewarding win or punishing loss) tends to bias accuracy in favour of the same context (e.g., more pronounced response to reward over punishment omission predicts higher accuracy in reward condition over punishment condition and vice versa). We also found that as Δpupil increases, Δaccuracy significantly decreases for positive‐outcome trials (Figure 2C; *R* ^2^ = 0.156, *F*(1,24) = 4.437, *p* = 0.046), but not for negative‐outcome trials (Figure 2D; *R* ^2^ = 0.001, *F*(1,24) = 0.028, *p* = 0.868). For positive outcomes, this suggests that relatively greater phasic arousal in response to wins reduces relative accuracy in the reward condition compared to the punishment condition, and vice versa. These findings show that, in line with our second aim, we are able to predict behavioural changes across context from EEG and pupil signals. It should be noted that the significant pupil result does not survive a Bonferroni correction for multiple comparisons, which lowers the alpha to 0.0125, demanding a level of caution for interpretation. All other results are unaffected.

Our final hypothesis proposed that the salience‐related EEG component would be related to pupil dilation, and that this relationship might offer further explanatory power in relation to behavioural changes across context. Given that pupil dilation is used here as a proxy for early LC arousal signals in the brainstem, and the projections that exist from LC to the regions associated with our early salience component in the EEG (e.g., Joshi and Gold 2022), we believe that the EEG component may reflect a downstream cortical salience representation of which is influenced by LC activation and subsequently drives behaviour. As such, given that both signals influence behaviour for positive outcomes, we believe that the EEG signals may be mediating an effect of LC arousal on behaviour. To reiterate the precondition checks, (1) the predictor variable (Δpupil) should significantly predict the outcome variable (Δaccuracy) in a simple linear regression; (2) the predictor variable (Δpupil) should significantly predict the mediator variable (Δ*Y*) in a simple linear regression; and (3) the mediator variable (Δ*Y*) should significantly predict the outcome variable (Δaccuracy) (Baron and Kenny 1986; Shrout and Bolger 2002). As with Δaccuracy, Δpupil was found to significantly predict Δ*Y* for positive‐outcome trials (Figure 3A; *R* ^2^ = 0.361, *F*(1,24) = 13.584, *p* = 0.001), but not for negative‐outcome trials (Figure 3B; *R* ^2^ = 0.056, *F*(1,24) = 1.414, *p* = 0.246), so the mediation analysis was only conducted for positive outcomes. The final bootstrapped comparison between the coefficient of Δpupil for predicting Δaccuracy with (*c′*) and without (*c*) Δ*Y* included as a predictor was significant (*p* < 0.001, Figure 3C,D), indicating that changes in pupil‐related arousal signals following positive outcomes may influence accuracy changes via distinct cortical activity across reward and punishment contexts.

### 3.4 Accuracy Effects (But Not EEG or Pupil) Are Predicted by Model‐Derived Choice Stochasticity

To further explore the nature of the context effects on choice accuracy, we compared differences across context in the free parameters of a reinforcement learning model with accuracy asymmetry. The learning rate reflects the weight applied to new information, and the slope—also known as the inverse temperature—reflects the degree of stochasticity or exploration in choice behaviour. As with our other measures, we computed a delta value for each by subtracting the value estimated from a model trained on punishment blocks from that of a model trained on reward blocks. Using a robust correlation (bendcorr: https://github.com/CPernet/Robust‐Correlations/blob/v2/bendcorr.m), we found that Δslope was significantly correlated with Δaccuracy (*r*(31) = 0.464, *p* = 0.006), but Δlrate was not (*r*(31) = −0.268, *p* = 0.131). This result suggests that reduction in accuracy going from one context to another tended to be driven by an increase in exploration and lower stability in symbol selection.

Further exploring the behavioural model parameters in relation to the EEG measures, we examined the relationship between Δslope and Δlrate and Δ*Y* for positive and negative outcomes separately, following a similar analysis strategy as in Figure 2 except using robust correlation instead of a linear model. Though trend‐level relationships were visible, there were no significant correlations between Δslope and Δ*Y* for positive (*r*(30) = 0.262, *p* = 0.147) or negative (*r*(30) = −0.288, *p* = 0.110) outcomes. This was also the case for Δlrate for both positive (*r*(30) = −0.315, *p* = 0.079) or negative (*r*(30) = 0.305, *p* = 0.089) outcomes. This suggests that while it is not implausible that the context sensitivity signals from the EEG analysis have some effect on choice stochasticity and rate of value updating, this is not enough to explain the strong accuracy asymmetry effects that we see in Figure 2A,B.

## 4 Discussion

In this study, we aimed to determine whether feedback in a punishing context elicits a distinct salience‐related signal when compared to a rewarding context. We show through multivariate discrimination analysis that EEG signals in response to punishment are highly separable from reward omission, and likewise for punishment avoidance and reward. By isolating an EEG signal that temporally coincides with a typical salience component of feedback processing (Fouragnan et al. 2015; Fouragnan, Retzler, and Philiastides 2018), we find distinct associations between mean discrimination amplitude and broad performance asymmetries across context. The phasic pupil responses to feedback were significantly amplified in the punishing context compared to the rewarding context, the magnitude of which also predicted performance differences, with a significant mediation effect of the EEG signal on this relationship. These findings suggest firstly that an initial salience response to feedback—possibly originating in the noradrenergic system in the brainstem—is modulated by an aversive context, and secondly that the degree to which this occurs has a significant direct effect on overall decision accuracy.

Given the absence of any direct group‐level effects of context on behaviour, the predictive strength of subject‐specific discrimination components on accuracy is notable. This finding demonstrates that the degree to which an individual reacts differently to rewarding and punishing stimuli at the neural level can reliably predict meaningful behavioural manifestation. Parallels can be drawn to certain biologically‐based theories of personality, such as reinforcement sensitivity theory (Corr 2004; Gray 1981; McNaughton and Corr 2008). This theory proposes that at the fundamental level, human behaviour is largely built upon innate sensitivity to different kinds of reinforcers, which manifest in distinct approach and avoidance behavioural systems (McNaughton and Corr 2008). The approach system largely overlaps with reward‐ and motivation‐related dopaminergic pathways including VTA and the vSTR (Depue and Collins 1999), whereas the avoidance system involves the amygdala and ACC among other arousal‐related regions (Corr 2004). This advance‐retreat dichotomy echoes the highly replicated finding that rewards are more associated with a ‘go’ response of behavioural invigoration (McNaughton and Gray 2000), and punishments are conversely associated with a ‘no‐go’ response of behavioural suppression.

Central to our hypothesis that individual differences in reinforcement sensitivity drive performance differences across contexts containing rewarding versus punishing reinforcers, we propose that the corresponding motivational asymmetry produces systematic differences in the motivational salience response to feedback. It has been shown that the mere possibility of receiving a rewarding outcome in a given environment can provoke a motivational salience response in dopaminergic regions to completely neutral stimuli (Kobayashi and Schultz 2014). Accordingly, altered motivational responses in the presence of potential rewards or punishments may lead to behavioural changes, such as a shift in exploration tendency (Blanchard, Griebel, and Blanchard 2001; J. Blanchard et al. 1998) or startle response (Aluja et al. 2015). Such behavioural shifts may affect task performance, bringing the agent closer to or further from optimal action, consistent with the strong link we showed between context differences in the EEG signal and overall choice accuracy.

To help contextualise the spatially integrated EEG signals from our multivariate discrimination output, we can look to widely studied event‐related‐potentials (ERPs) that show spatio‐temporal and theoretical similarity with our early component. Prior work with a similar two‐component EEG analysis as the present study has shown a notable link between the early salience component and the feedback‐related negativity (FRN) ERP (Philiastides et al. 2010). This tracks with subsequent EEG‐fMRI analyses that found the ACC to be strongly implicated in the same early component (Fouragnan et al. 2015)—a region in which the FRN is typically source localised (Walsh and Anderson 2012). The typical temporal range appearing in FRN research is 200–300 ms (Cohen, Wilmes, and van de Vijver 2011; Holroyd and Coles 2002; van de Vijver, Ridderinkhof, and Cohen 2011), with many studies finding peak FRN responses in the early portion of this range within 5–10 ms of our discrimination peaks (e.g., Hauser et al. 2014; Philiastides et al. 2010; Talmi, Atkinson, and El‐Deredy 2013), and the primary electrode used in FRN analyses (FCz) lies directly in the centre of our frontal topographical clusters (Figure 1C).

Though initially proposed to reflect a direct RPE signal (Bellebaum, Polezzi, and Daum 2010; Chase et al. 2011; Holroyd and Coles 2002), a growing body of research has challenged this view of the FRN with evidence that it better reflects a ‘good versus bad’ outcome valence signal that is distinct from value or surprise (Fouragnan, Retzler, and Philiastides 2018; Hajcak et al. 2006; Philiastides et al. 2010; Sato et al. 2005; Toyomaki and Murohashi 2005; Yeung and Sanfey 2004). This has also been characterised explicitly as a motivational salience signal common across rewarding and aversive stimuli (Mason et al. 2016; Talmi, Atkinson, and El‐Deredy 2013), a view consistent with findings that an active rather than passive learning enhances FRN magnitudes, implying that motivational relevance is a key element of the signal (Itagaki and Katayama 2008; Marco‐Pallarés et al. 2010; Martin and Potts 2011; Yeung, Holroyd, and Cohen 2005). The distinction from surprise is also consistent with our findings that unsigned prediction errors do not significantly explain variance in our weighted EEG signal (Figure 1D). There is also a body evidence linking FRN responses and external measures of punishment sensitivity (Balconi and Crivelli 2010; De Pascalis, Varriale, and D'Antuono 2010; Massar et al. 2012; Santesso, Dzyundzyak, and Segalowitz 2011; Unger, Heintz, and Kray 2012). We do not suggest that our spatially weighted EEG signal is completely analogous to the FRN, which are typically reported from individual sensors of interest. However, we believe there is enough conceptual and spatio‐temporal overlap to consider this a useful known signal that can offer insight into the makeup of our discrimination component.

Consistent with the proposed role of a motivational salience response in differentiating reward and punishment learning, the early component is also strongly linked with the aINS and amygdala in both rewarding (Carvalheiro and Philiastides 2023; Fouragnan et al. 2015, 2017) and punishing contexts (Carvalheiro and Philiastides 2023). Though active in both contexts, these two regions have been implicated repeatedly in a specific capacity within punishment learning (Palminteri and Pessiglione 2017). Activity in the aINS has been directly related to a computational PPE (Kim, Shimojo, and O'Doherty 2006; Seymour et al. 2004; Skvortsova, Palminteri, and Pessiglione 2014), while damage to the amygdala is known to inhibit salience processing of arousing stimuli (e.g., Anderson and Phelps 2001) as well as punishment learning (Bechara et al. 1995; De Martino, Camerer, and Adolphs 2010). The specific role in punishment learning combined with the presence in the early component of reward learning suggest that these regions could house motivational salience signals that are asymmetrically sensitive to appetitive and aversive reinforcers.

We showed that differences in pupil dilation in response to rewards versus punishment omissions seem to strongly predict the corresponding differences in weighted EEG signal (Figure 3A), and moderately track accuracy asymmetries (Figure 2C). Given that noradranergic LC activation is known to drive phasic pupil dilation (Larsen and Waters 2018; Mathôt 2018), we interpret these signals conservatively as an indirect proxy for activity in this nucleus. The LC has noradrenergic projections to both the amygdala (Buffalari and Grace 2007; McCall et al. 2017) and the ACC (Carvalheiro and Philiastides 2023; Chandler and Waterhouse 2012; Hamner, Lorberbaum, and George 1999; Joshi and Gold 2022; Koga et al. 2020), and these projections are implicated in alertness and attention (Sara 2009; Sara and Bouret 2012), which we use as the basis for a possible early arousal signal propagating from the LC to influence salience processing from outcomes. Though it has been shown that cortical signals which occur after our early EEG component, such as the P3 ERP, can exhibit a relationship with the phasic pupil response, these signals are generally believed to be co‐generated alongside pupil dilation by noradrenergic LC signals (Chang et al. 2024; Menicucci et al. 2024; Nieuwenhuis 2011; Nieuwenhuis, Aston‐Jones, and Cohen 2005). This also accounts for cases where the P3 and pupil dilations were found to be uncorrelated (De Gee et al. 2021; Kamp and Donchin 2015; LoTemplio et al. 2021), and we believe that these findings are in line with the proposed mediation pathway from LC to cortex to behaviour that we propose in our results.

It important to note that the pupil effects from our data were not present for negative outcomes—the reward omission versus punishment comparisons. Since negative outcomes were less frequent (therefore more surprising) and provoked a much larger pupil response on average (Figure  1A,B), we speculate that this is due to a ceiling effect of pupil diameter, whereby more subtle changes across context are less detectable as the pupil nears maximum dilation. Recent work has shown that LC activity differs significantly between positive and negative outcomes in a rewarding context but not in a punishing context (Carvalheiro and Philiastides 2023), which seems consistent with the idea that LC activity is higher across the board in a punishing context and perhaps therefore less differentiable, as indicated by the broadly higher dilation we observe. However, this hypothesis has not been directly tested and remains conservative.

In addition to areas in the cortical salience network, the LC also projects to the habenula (Purvis, Klein, and Ettenberg 2018; Root et al. 2015), which has been directly implicated in the processing of motivational salience (Bromberg‐Martin, Matsumoto, and Hikosaka 2010a; Danna, Shepard, and Elmer 2013; Fakhoury and Domínguez López 2014; Hikosaka 2010) as well as aversive stimuli (Hennigan, D'Ardenne, and McClure 2015; Lawson et al. 2014; Lecca et al. 2017; Mondoloni, Mameli, and Congiu 2022). This is relevant to one of the main hypotheses of outcome encoding in punishment learning—that punishment is encoded with firing dips in midbrain dopaminergic neurons (Matsumoto and Hikosaka 2009)—as it provides a possible route from early outcome‐driven arousal signals in the LC to the encoding of reward and punishment in the VTA and substantia nigra via inhibitory signals from the habenula (Christoph, Leonzio, and Wilcox 1986; Hikosaka 2010; Matsumoto and Hikosaka 2007). This provides further support for the plausibility of our proposed mediation pathway, although we reiterate that this would require further research to test.

**FIGURE 7:**
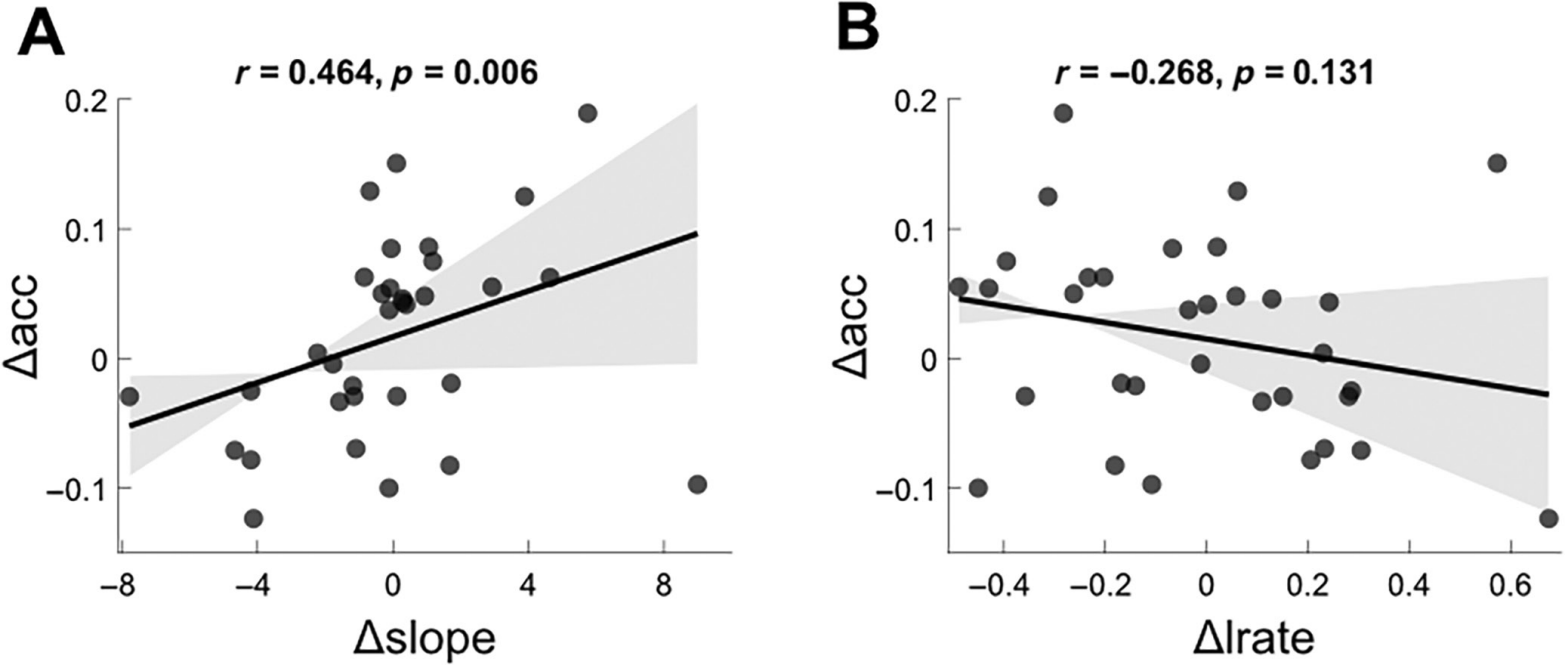
Accuracy asymmetry correlated with differences in slope and learning rate. (A) Change in slope (reward–punishment) on the *x*‐axis significantly correlates with change in accuracy on the *y*‐axis, but (B) change in learning rate does not.

**FIGURE 8:**
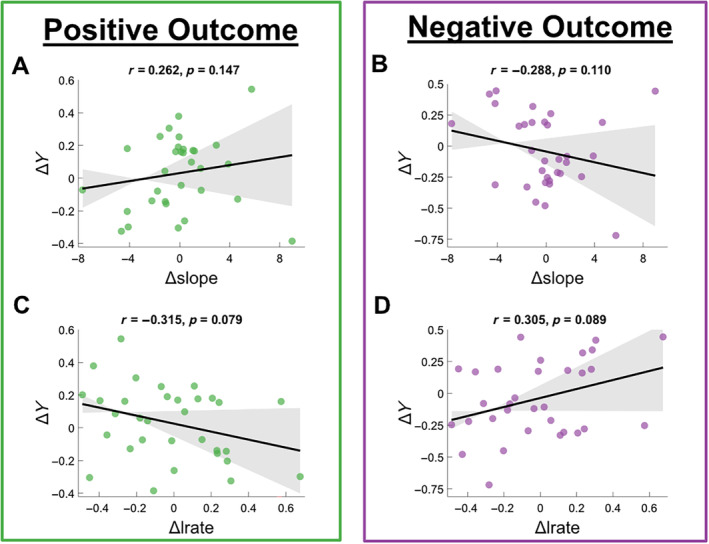
(A and B) Δ*Y* correlated with Δslope across subjects. Shaded error bars indicate 95% confidence intervals for the estimate. Δ*Y* is separated by classification model trained on positive‐outcome (left, green) and negative‐outcome (right, purple) trials. Δslope is identical across both plots. Positive value on *y*‐axis indicates that EEG data for reward condition is on average further from the discriminating hyperplane than EEG data for punishment condition in a given participant. Positive value on the *x*‐axis indicates lower choice stochasticity in reward condition versus punishment condition for a given participant. (C and D) Equivalent plots with Δlrate depicted on the *x*‐axis rather than Δslope. Δlrate is identical across both plots, whereas Δ*Y* is separated by outcome type. Positive value on the *x*‐axis indicates higher weight applied to incoming decision outcomes in the reward condition versus punishment condition for a given participant.

As with unsigned prediction errors (or surprise), there were no significant relationships across subjects between our weighted EEG signal and the inverse temperature (slope) and learning rate parameters from our reinforcement learning model (Figure 7A–D). This is somewhat surprising, as inverse temperature—which can be conceptualised as the rate of stochasticity in choice behaviour—was highly predictive of accuracy asymmetries (Figure 8A), as was the EEG component itself (Figure 2A,B). This seems to suggest that although increases in EEG amplitude from one context to another weakly track corresponding increases in choice randomness and exploration at a non‐significant trend level, there seem to be other processes contained in the weighted EEG signal that reduce accuracy in a non‐systematic manner. It is important to note that the direction of these context effects are heterogeneous across subjects, in the sense that some subjects experience accuracy reduction in the punishment context, while others see an enhancement (Figure 6A). This could imply a myriad of possible interactions between a context‐related motivational salience response and downstream behavioural effects such as a helpful enhancement of memory and focus (e.g., Sutherland and Mather 2015, 2018), or an unhelpful dysregulated arousal response to non‐salient or neutral motivational stimuli that could be exacerbated, for example, in cases of anxiety or schizophrenia (Neumann, Glue, and Linscott 2021).

Accordingly, the link between neural differences during reward versus punishment processing and subsequent behaviour may have important applications in clinical settings. For instance, disorders characterised by elevated DA in fronto‐striatal regions typically predict deficits in punishment learning and behavioural inhibition, including schizophrenia (Moustafa et al. 2015) and Tourette's syndrome (Palminteri et al. 2012). Conversely, patients with Parkinson's disease and major depressive disorder can experience severe motivational apathy, largely attributed to a deficit in fronto‐striatal DA (Pagonabarraga et al. 2015) and blunted RPE responses in the striatum and amygdala (Queirazza et al. 2019), respectively. These individuals also show significantly reduced distinction in neural response to outcome valence (e.g., gains versus losses), characterised by changes in FRN amplitudes (Martínez‐Horta et al. 2014). Here, we have identified a similar neural signature predicting interindividual behavioural performance across outcome types—in the absence of group‐level trends—which could be used as a proxy for a more targeted diagnostic stratification and a more individualised treatment planning.

## Conflicts of Interest

The authors declare no conflicts of interest.

## Data Availability

The data that support the findings of this study are openly available in the Open Science Framework (OSF) at https://osf.io/5zj2s/.
